# REMS versus DXA in older adults: diagnostic concordance for osteoporosis in a geriatric outpatient population

**DOI:** 10.1007/s11657-026-01723-8

**Published:** 2026-06-11

**Authors:** Chiara Ceolin, Marianna Noale, Cristina Simonato, Franz Villanova, Silvia Sturani, Anna Bertocco, Sandro Giannini, Gaetano Paride Arcidiacono, Luca Busetto, Angelo Antonini, Alessandra Coin, Giuseppe Sergi, Marina De Rui

**Affiliations:** 1 Department of Medicine (DIMED), University of Padua, Padua, Italy; 2 Geriatric Unit, University Hospital of Padua, Padua, Italy; 3 Department of Neurobiology, Care Sciences and Society, Aging Research Center, Karolinska Institutet and Stockholm University, Stockholm, Sweden; 4 National Research Council, Neuroscience Institute, Padua, Italy; 5 Clinica Medica 1, University Hospital of Padua, Padua, Italy; 6 Clinical Nutrition Unit, University Hospital of Padua, Padua, Italy; 7 Neurodegenerative Disease Unit, Department of Neuroscience, Padua Neuroscience Center (PNC), University of Padua, Padua, Italy; 8 IRCCS San Camillo, Venice, Italy

**Keywords:** Radiofrequency echographic multispectrometry (REMS), Dual-energy X-ray absorptiometry (DXA), Osteoporosis, Bone mineral density, Older adults, Diagnostic agreement

## Abstract

***Summary*:**

REMS showed moderate concordance with DXA but limited interchangeability in geriatric outpatients. Its high negative predictive value supports its use as a practical first-line screening method, especially in adults ≥ 75 years. Findings highlight REMS’ potential in settings where DXA is limited and the need for further validation.

**Purpose:**

Concordance between dual-energy X-ray absorptiometry (DXA) and radiofrequency echographic multispectrometry (REMS) in older adults is heterogeneous, and their interchangeability at the patient level is uncertain. The objective of this study is to examine correlations, diagnostic agreement, and performance of REMS versus DXA in geriatric outpatients, focusing on individuals aged ≥ 75 years and BMI.

**Methods:**

Cross-sectional study of 148 outpatients (median age 75 [70–79] years, 55.4% women). DXA and REMS were performed at the lumbar spine, total hip, and femoral neck. We calculated Spearman correlations, diagnostic performance of REMS using DXA as reference, categorical agreement (Cohen’s kappa), linear regressions, and Bland–Altman plots, stratified by age (< 75/ ≥ 75 years) and BMI.

**Results:**

DXA–REMS correlations were moderate overall (*ρ* 0.52–0.64 for BMD and *T*-scores) but stronger in participants ≥ 75 years (up to *ρ* 0.75–0.78 at the total hip, *p* < 0.001). REMS sensitivity was 46–62%, specificity 72–79%, PPV 0.23–0.40, and NPV 0.82–0.93 for osteoporosis (kappa 0.15–0.27). Bland–Altman analyses showed wide limits of agreement, indicating poor interchangeability at the individual level. In adults ≥ 75 years, REMS yielded lower BMD and more negative *T*-scores at all sites, whereas DXA did not differ by age group. Low BMI was consistently associated with lower BMD and more negative *T*-scores.

**Conclusions:**

REMS shows moderate concordance with DXA and limited interchangeability, but its high NPV supports a pragmatic role as a first-line screening tool in geriatric practice, especially in adults ≥ 75 years and when DXA is unavailable.

**Supplementary Information:**

The online version contains supplementary material available at 10.1007/s11657-026-01723-8.

## Introduction

Accurate diagnosis of osteoporosis in older adults remains a clinical challenge. Dual-energy X-ray absorptiometry (DXA) is widely regarded as the reference method for assessing bone mineral density (BMD) at the spine and hip [1, 2], yet its performance in geriatric practice is far from ideal [3]. Paradoxically, it is in older adults—the population most exposed to fracture risk—that diagnostic accuracy is most uncertain. Age-related degenerative changes such as osteoarthritis, vertebral deformities, and abdominal aortic calcifications frequently introduce artifacts, leading to an overestimation of bone density, especially at the lumbar spine [4–6]. Metallic implants and prostheses, increasingly common in this age group, may further limit the feasibility of measurement at key skeletal sites [7, 8]. In addition to these technical challenges, DXA entails exposure to ionizing radiation and is affected by operator-dependent variability [9, 10]. Notably, guideline deviations have been documented in the majority of scans, raising concerns about the reliability of routine reports [1, 11, 12]. Collectively, these limitations may undermine the diagnostic value of DXA precisely in the population where accurate detection of osteoporosis is most crucial.

Radiofrequency echographic multispectrometry (REMS) has recently emerged as an alternative that may overcome several of these barriers. REMS is a radiation-free, ultrasound-based technique that acquires raw radiofrequency signals at the lumbar spine and femoral neck, subsequently processed through spectral analysis algorithms to yield site-specific BMD values and *T*-scores [13]. REMS is portable, fast (less than 2 min per site including automated processing), and relatively inexpensive [14, 15]. Importantly, multicenter validation studies have demonstrated strong correlations with DXA, good diagnostic accuracy, and predictive value for fracture risk, with international consensus statements endorsing its potential role in clinical practice [16, 17]. Yet, despite encouraging validation data, most prior investigations have been limited to postmenopausal women, often excluding frail or clinically complex individuals. Evidence on REMS performance in geriatric outpatients—who typically present multiple comorbidities, high prevalence of degenerative changes, and frequent mobility limitations—is scarce. This population represents a crucial testing ground for REMS, precisely because it is here that the limitations of DXA are most evident and the need for radiation-free, bedside-capable alternatives is greatest.

## Purpose

Given these premises, the primary objective of this study was to assess the concordance between REMS and DXA in geriatric outpatients by quantifying (i) correlations for BMD and *T*-scores at major skeletal sites and (ii) categorical diagnostic agreement (osteoporosis/osteopenia/normal), including Bland–Altman analyses to evaluate patient-level interchangeability. Secondary objectives were (i) to evaluate the diagnostic performance of REMS against DXA as reference (sensitivity, specificity, positive predictive value (PPV), and negative predictive value (NPV)) for osteoporosis classification; (ii) to examine the impact of advanced age (considering 75 years as a cut-off derived from the median age value of our sample), on the REMS–DXA relationship; and (iii) to investigate the association of BMI with BMD and *T*-scores measured by both techniques.

## Materials and methods

### Study design and population

This retrospective study is part of the OPA (obesity, Parkinson’s disease, and Alzheimer’s disease biomarkers of aging) project, an observational cohort study conducted between September 2023 and June 2025. In addition to participants enrolled in the OPA study, we also included consecutively recruited older outpatients evaluated during the same period. Overall, we enrolled community-dwelling individuals aged ≥ 65 years consecutively attending the Geriatrics, Neurology, or Internal Medicine units of the University Hospital of Padua (Italy). Inclusion criteria included the ability to understand instructions and correctly perform the proposed tests. Exclusion criteria were acute illness, severe dehydration, decompensated heart failure with edema, unstable cardiovascular or metabolic conditions (e.g., uncontrolled arrhythmias, hypertension, recent myocardial infarction, or severe dementia), and hospitalization within the previous 3 months.

The study protocol adhered to the principles of Good Clinical Practice and the ethical standards of the 1964 Declaration of Helsinki and its 2000 revision. Ethical approval (protocol no. 5707/AO/23) was granted by the Clinical Research Ethics Committee of the Province of Padua, Italy. Written and verbal informed consent was obtained from all participants after a detailed explanation of the risks and benefits associated with participation.

### Data collection

#### Patient characteristics

Demographic, clinical, and pharmacological data were collected through structured interviews conducted by trained physicians. Information included a comprehensive geriatric assessment. Comorbidities were assessed using the Cumulative Illness Rating Scale (CIRS) [18]. Functional autonomy was measured with the Activities of Daily Living (ADL) scale [19]. Nutritional status was evaluated using the Mini Nutritional Assessment (MNA) [20].

#### Anthropometric measurements

Body weight was measured using a calibrated digital scale (accuracy 0.1 kg). Standing height was estimated using knee-heel length according to Chumlea’s equations in nonambulatory participants. Body mass index (BMI) was calculated as weight in kilograms divided by height in meters squared.

#### Bone assessment

All participants underwent DXA scans (Hologic QDR 4500 W, fan-beam technology) to assess BMD at the proximal femur (femoral neck and/or total hip) and lumbar spine. Instrument performance was ensured through routine phantom calibration, and precision testing was conducted according to ISCD recommendations. Bone status was additionally assessed using REMS, a noninvasive, radiation-free ultrasound technique (EchoStation, Echolight S.p.A., Lecce, Italy). REMS acquisitions were performed at the lumbar spine and proximal femur (femoral neck and total hip) by trained operators, following manufacturer’s recommendations and international guidelines. Briefly, raw backscattered ultrasound signals were collected through a convex probe and processed by a spectral analysis algorithm that identifies and isolates bone-specific patterns from soft tissue and artifactual components. From the validated spectral models, the device provides quantitative estimates of bone mineral density (BMD, g/cm^2^) and corresponding *T*-scores. Participants were examined in the supine position, with the transducer positioned over the target skeletal site using anatomical landmarks and real-time ultrasound guidance. At least two valid acquisitions were required at each site; scans were repeated if motion artifacts or suboptimal positioning were detected. Measurements were automatically averaged by the software to generate site-specific BMD and *T*-score values. DXA scans were acquired and interpreted according to routine clinical practice, following WHO/ISCD-based standards. Lumbar spine measurements were obtained from postero-anterior L1–L4 projections. Vertebrae affected by local structural changes or artifacts (e.g., osteoarthritis, vertebral deformities, or calcifications) were excluded from analysis when appropriate, and lumbar *T*-scores were calculated from the remaining evaluable vertebrae, ensuring a sufficient number of vertebrae for valid diagnosis according to ISCD criteria. DXA and REMS examinations were performed by two adequately trained operators; the same operator conducted both examinations for each participant to minimize interoperator variability.

### Definition of osteoporosis

Bone health categories were defined according to WHO criteria [21] and interpreted in accordance with current ISCD positions. In postmenopausal women and men aged ≥ 50 years, osteoporosis was defined as a *T*-score ≤  − 2.5 at the lumbar spine, total hip, or femoral neck. For hip *T*-scores, the reference population was the manufacturer-implemented NHANES III young-adult female database, whereas lumbar spine *T*-scores were derived using the manufacturer’s reference database, as recommended by ISCD. The same diagnostic thresholds were applied to REMS-derived *T*-scores to allow direct comparison between the two techniques.

### Statistical analysis

We categorized BMI as low (< 24.1 kg/m^2^), moderate (24.2–27.1 kg/m^2^), and high (≥ 27.1 kg/m^2^) according to tertile distributions. Moreover, according to the median value of age, participants were stratified into two age groups: < 75 years and ≥ 75 years. Baseline characteristics of participants, stratified by BMI classes or age groups, were described as mean and standard deviation (SD), or median and interquartile ranges (IQR) for quantitative variables, and as counts and percentages for categorical variables. Normal distributions of continuous variables were tested using the Kolmogorov–Smirnov test. Group comparisons were performed using the Student *t*-test for normally distributed continuous variables, the Kruskal–Wallis test for nonnormally distributed variables, and the chi-square test (or Fisher’s exact test when appropriate) for categorical variables. Correlations between DXA and REMS bone parameters were assessed using Pearson or Spearman correlation coefficients, depending on variable distribution.

Accuracy of REMS was evaluated considering DXA outputs as the gold standard reference. Diagnostic accuracy of REMS was assessed by calculating sensitivity, specificity, PPV, and NPV for osteoporosis classification at the total hip, femoral neck, and lumbar spine, for both BMD and *T*-score measures. Chi-square tests assessed differences in classification distributions. Agreement between REMS and DXA *T*-score classifications was quantified using Cohen’s kappa coefficient with corresponding 95% confidence intervals (CIs) to account for chance agreement. Linear regression models were fitted to quantify systematic differences between DXA and REMS, with slopes and intercepts reported; coefficient of determination (*R*^2^) was also evaluated to assess the proportion of the variance in the dependent variable that was predictable from the independent variables in the linear model. Bland–Altman analyses were conducted to evaluate measurement bias and limits of agreement between the two methods. Continuous agreement between REMS and DXA, also Lin’s concordance correlation coefficient (CCC) was calculated for BMD and *T*-scores at each site, reflecting both precision (correlation) and accuracy (departure from the line of identity), and was computed from the mean, variance, and covariance of the paired measures.

No a priori sample size calculation was performed, and post hoc power was conducted, considering as primary endpoint for power assessment the correlation between REMS and BMD measurements. Considering the final sample size (148 subjects), the minimum detectable correlation with 80% power (two-sided *α* = 0.05) was 0.23, indicating that the study was adequately powered to detect moderate to strong associations (≥ 0.30).

Statistical significance was set at *p* < 0.05. All analyses were performed using SAS 9.4. (Cary, NC) statistical package and IBM SPSS Statistics for Windows, Version 29.0 (IBM Corp., Armonk, NY, USA).

## Results

### Baseline characteristics

Of the 230 individuals initially enrolled, participants with incomplete or missing DXA or REMS measurements were excluded. The final analytic sample therefore consisted of 148 older adults [median age 75 (70–79) years, 55.4% female]. Descriptive characteristics of the study population, stratified by BMI classes, are summarized in Table 1. No significant differences were observed in age, sex distribution, functional independence, comorbidity burden, or number of medications across BMI groups (all *p* > 0.05). However, nutritional status differed significantly between groups (*p* = 0.02), with the moderate BMI group demonstrating higher scores compared to the low and high BMI groups. Bone health, assessed by both DXA and REMS, showed consistent patterns. Participants with low BMI exhibited significantly lower BMD values at all sites (lumbar spine, total hip, and femoral neck) and more negative *T*-scores compared to those with moderate and high BMI (all *p* < 0.001). This trend was evident in both DXA and REMS-derived measurements. Considering age groups, individuals aged ≥ 75 years showed significantly lower functional independence and a higher burden of comorbidities (please see Online resource-Supplementary Table 1). Analysis of BMD and *T*-scores using DXA did not reveal significant differences between age groups across the lumbar spine, total hip, or femoral neck regions. In contrast, when measured by REMS, BMD values at all skeletal sites were significantly lower in the older age group (all *p* ≤ 0.003). Correspondingly, REMS *T*-scores were more negative in the ≥ 75 group, with statistically significant differences for all skeletal sites.

**Table 1:** Baseline characteristics of the study population stratified by BMI categories

Variable	All sample (*n* = 148)	Low BMI (*n* = 39)	Moderate BMI (*n* = 66)	High BMI (*n* = 43)	*p* value
Age (years)	75 (70, 79)	75 (69, 78)	74 (70, 81)	75 (70, 80)	0.75
Sex, females	82 (55.4%)	24 (61.5%)	31 (47.0%)	27 (62.8%)	0.18
ADL	6 (5, 6)	6 (5, 6)	6 (5, 6)	6 (5, 6)	0.45
CIRS-CI	1 (1, 2)	1 (1, 2)	1.55 (1.00, 2.00)	2 (1, 3)	0.12
MNA	25.50 (24.00, 27.50)	25.00 (23.50, 26.00)	26.50 (24.50, 28.00)	25.00 (23.00, 27.00)	0.02
Total no. drugs	3 (1, 5)	2 (1, 5)	3 (1, 5)	4 (2, 6)	0.09
*Bone parameters (DXA)*					
BMD lumbar	0.99 (0.84, 1.12)	0.84 (0.74, 0.96)	1.02 (0.87, 1.11)	1.05 (0.94, 1.23)	< 0.001
BMD total hip	0.82 (0.71, 0.95)	0.73 (0.67, 0.84)	0.83 (0.72, 0.93)	0.95 (0.78, 1.03)	< 0.001
BMD femur neck	0.68 (0.59, 0.79)	0.61 (0.56, 0.69)	0.69 (0.59, 0.78)	0.75 (0.64, 0.86)	< 0.001
*T*-score lumbar	−0.60 (−1.90, 0.40)	−1.80 (−2.80, −0.80)	−0.40 (−1.55, 0.40)	0 (−1.00, 1.60)	< 0.001
*T*-score total hip	−1.20 (−2.00, −0.50)	−1.80 (−2.40, −1.20)	−1.10 (−1.92, −0.50)	−0.50 (−1.33, 0.33)	< 0.001
*T*-score femur neck	−1.70 (−2.40, −1.10)	−2.20 (−2.60, −1.60)	−1.70 (−2.42, −1.05)	−1.15 (−1.83, −0.38)	< 0.001
*Bone parameters (REMS)*					
BMD lumbar	0.85 (0.78, 0.91)	0.80 (0.75, 0.81)	0.85 (0.77, 0.89)	0.94 (0.87, 1.00)	< 0.001
BMD total hip	0.73 (0.64, 0.81)	0.62 (0.58, 0.67)	0.73 (0.67, 0.78)	0.85 (0.77, 0.90)	< 0.001
BMD femur neck	0.62 (0.54, 0.70)	0.52 (0.48, 0.56)	0.62 (0.60, 0.67)	0.72 (0.65, 0.77)	< 0.001
*T*-score lumbar	−2.05 (−2.60, −1.40)	−2.60 (−2.93, −2.40)	−2.10 (−2.60, −1.80)	−1.30 (−1.70, −0.60)	< 0.001
*T*-score total hip	−1.90 (−2.50, −1.40)	−2.70 (−3.00, −2.40)	−1.90 (−2.30, −1.60)	−1.00 (−1.40, −0.60)	< 0.001
*T*-score femur neck	−2.30 (−2.80, −1.70)	−3.00 (−3.30, −2.60)	−2.30 (−2.60, −1.90)	−1.40 (−1.90, −0.90)	< 0.001

Numbers are presented as median (IQR), or count (percentage), as appropriate

*BMI* body mass index, *ADL* activities of daily living, *CIRS-CI* cumulative illness rating scale–comorbidity index, *MNA* mini nutritional assessment, *BMD* bone mineral density

### Correlation tests

Spearman correlation analyses revealed moderate to strong positive correlations between bone parameters assessed by DXA and those assessed by REMS across the entire sample. Specifically, DXA-derived lumbar BMD was significantly correlated with REMS lumbar BMD (*ρ* = 0.52, *p* < 0.001), and similar results were observed for femoral total BMD (*ρ* = 0.64, *p* < 0.001) and femoral neck BMD (*ρ* = 0.55, *p* < 0.001). Strong correlations were also found between DXA *T*-scores and REMS *T*-scores at corresponding anatomical sites.

**Fig. 1:**
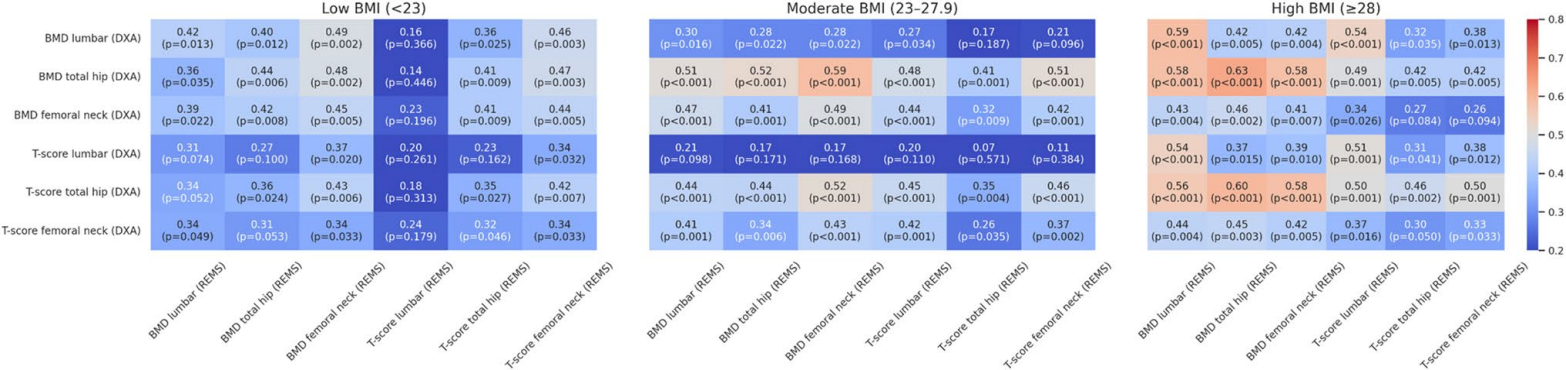
Correlation between DXA and REMS parameters according to BMI classes. Abbreviations: BMI: body mass index; BMD: bone mineral density

In participants with low BMI, significant positive correlations were observed between most DXA and REMS parameters, particularly between femoral total BMD (DXA) and total hip BMD (REMS) (*ρ* = 0.44, *p* = 0.006), and between femoral neck *T*-score (DXA) and femoral neck *T*-score (REMS) (*ρ* = 0.43, *p* = 0.007). However, several correlations did not reach statistical significance, especially those involving lumbar *T*-scores (REMS) (Fig. 1). In the moderate BMI group, femoral total BMD (DXA) showed high correlation with total hip BMD (REMS) (*ρ* = 0.59, *p* < 0.001), and femoral neck BMD (DXA) was significantly correlated with all REMS femoral neck measures. *T*-score lumbar (DXA) showed no correlations with REMS lumbar parameters. Among participants with high BMI, correlations were generally stronger and more homogeneous. For instance, lumbar BMD (DXA) was significantly correlated with lumbar BMD from REMS (*ρ* = 0.59, *p* < 0.001), and femoral total *T*-score (DXA) showed robust associations with total hip *T*-score (REMS) (*ρ* = 0.50, *p* = 0.001) and femoral neck *T*-score (REMS) (*ρ* = 0.50, *p* = 0.001). Lumbar and femoral BMDs demonstrated the most consistent and statistically significant relationships across all REMS parameters.

When stratifying by age, some differences emerged (Online resource-Supplementary Fig. 1). In participants aged < 75 years, correlations were generally lower, particularly for femoral neck BMD and *T*-scores, where the coefficients ranged from *ρ* = 0.28 to 0.49. In this younger subgroup, the correlation between DXA and REMS lumbar *T*-score was *ρ* = 0.42 (*p* < 0.001), and the strongest correlation was observed for total hip *T*-score (*ρ* = 0.62, *p* < 0.001). In contrast, participants aged ≥ 75 years showed consistently stronger correlations across all measures, with coefficients reaching up to *ρ* = 0.78 for total hip BMD and *ρ* = 0.75 for total hip *T*-score (both *p* < 0.001). When stratifying by sex, significant positive correlations (*p* < 0.001) between REMS and DXA parameters were found in both males and females (Online resource-Supplementary Fig. 2), with consistently moderate-to-strong coefficients across all skeletal sites and *T*-scores, indicating a comparable performance of REMS in both sexes and a stable concordance with DXA measurements.

### Diagnostic performance of REMS

According to REMS, osteoporosis was identified in 40 patients at the total hip, 40 at the femoral neck, and 46 at the lumbar spine. In contrast, DXA classified 17 patients as osteoporotic at the total hip, 35 at the femoral neck, and 22 at the lumbar spine. Regarding the diagnostic performance of REMS for osteoporosis detection compared to DXA, sensitivity analyses revealed moderate detection capabilities: 53% at the total hip, 46% at the femoral neck, and 62% at the lumbar spine. These values indicate that REMS fails to identify approximately half of the true positive cases detected by DXA at femoral sites but performs somewhat better at the lumbar spine. Specificity was consistently higher, ranging from 72 to 79%, highlighting that REMS more reliably identifies nonosteoporotic subjects, which is clinically valuable for ruling out disease and reducing false positives (Table 2). PPVs ranged from 0.23 at the total hip to 0.40 at the femoral neck, which are relatively low, suggesting limited ability of REMS to confirm osteoporosis diagnosis when a positive result is obtained. In contrast, NPVs between 0.82 and 0.93 indicate strong confidence in REMS’s negative results. Chi-square tests showed statistically significant correlations between REMS and DXA osteoporosis classifications (*p* = 0.003–0.011), supporting the consistency of REMS categorizations with the gold standard despite moderate accuracy (Table 2). When considering the lowest *T*-score, REMS showed improved performance, with sensitivity increasing to 77% and NPV remaining high (0.86), although specificity decreased to 70% and PPV remained modest (0.55). These findings suggest that using the lowest site improves REMS’s ability to detect osteoporosis but still results in a meaningful proportion of false positives. Chi-square tests showed statistically significant associations between REMS and DXA osteoporosis classifications across all sites, supporting the consistency of REMS categorizations with the gold standard despite its overall moderate diagnostic accuracy (Table 2).

**Table 2:** Diagnostic performance of REMS for osteoporosis classification using DXA as reference (sensitivity, specificity, and predictive values)

		***Total hip***		
		**DXA**		
		**No osteoporosis**	**Osteoporosis**	
**REMS**	**No osteoporosis**	99 (92.5)	8 (7.5)	*χ*^2^ 6.43, *p* value = 0.0112
	**Osteoporosis**	31 (77.5)	9 (22.5)	Sensitivity 0.53
				Specificity 0.76
				Predictive positive value 0.23
				Predictive negative value 0.93
		***Femoral neck***		
		**DXA**		
		**No osteoporosis**	**Osteoporosis**	
**REMS**	**No osteoporosis**	88 (82.2)	19 (17.8)	*χ*^2^7.94, *p* value = 0.0048
	**Osteoporosis**	24 (60.0)	16 (40.0)	Sensitivity 0.46
				Specificity 0.79
				Predictive positive value 0.40
				Predictive negative value 0.82
		***Total lumbar***		
		**DXA**		
		**No osteoporosis**	**Osteoporosis**	
**REMS**	**No osteoporosis**	86 (91.5)	8 (8.5)	*χ*^2^ 9.06, *p* value = 0.0026
	**Osteoporosis**	34 (72.3)	13 (27.7)	Sensitivity 0.62
				Specificity 0.72
				Predictive positive value 0.28
				Predictive negative value 0.91
		***Lowest T-score***		
		**DXA**		
		**No osteoporosis**	**Osteoporosis**	
**REMS**	**No osteoporosis**	70 (86.4)	11 (13.6)	*χ*^2^ 29.02, *p* value < 0.0001
	**Osteoporosis**	30 (44.8)	37 (55.2)	Sensitivity 0.77
				Specificity 0.70
				Predictive positive value 0.55
				Predictive negative value 0.86

Agreement analyses using Cohen’s kappa statistic demonstrated low-to-moderate concordance between REMS and DXA categorical *T*-score classifications: 0.27 for the femoral neck, 0.24 for the total hip, and 0.15 for the lumbar spine. These kappa values, although statistically significant, reflect only fair agreement, emphasizing limitations in interchangeability when diagnosing osteoporosis categories (Table 3).

**Table 3:** Agreement between DXA- and REMS-derived categorical *T*-score classifications (osteoporosis/osteopenia/normal)

	Cohen’s kappa (95% CI)
Total hip	0.24 (0.13, 0.34)
Femoral neck	0.27 (0.14, 0.39)
Lumbar	0.15 (0.06, 0.24)

**Fig. 2:**
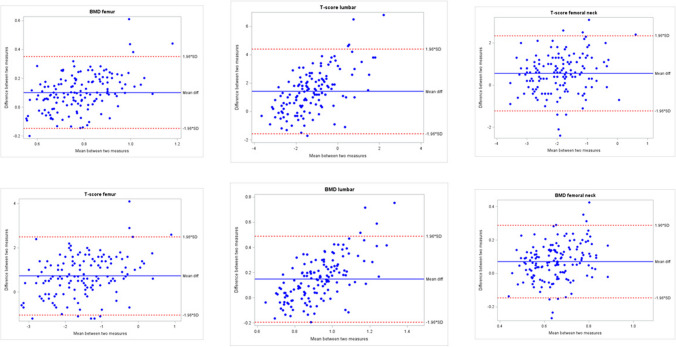
Bland–Altman plot comparing REMS and DXA BMD and *T*-score measurements. The difference between methods was defined as DXA minus REMS and plotted against the mean of the two techniques. Abbreviations: BMD: bone mineral density

Linear regression parameters exhibited slopes close to but generally below unity (0.60–1.04) and small intercepts, indicating minor systematic biases in REMS readings (Table 4). For example, the total hip BMD regression slope of 0.93 and intercept of 0.15 suggest a slight underestimation by REMS relative to DXA. Bland–Altman analyses corroborated these systematic differences, showing small mean differences (e.g., 0.10 ± 0.13 for total hip BMD) but with noticeable variability in agreement limits, indicative of measurement inconsistency at the individual level (Fig. 2). Furthermore, Bland–Altman plots showed a proportional bias at the lumbar spine: The difference between DXA and REMS increased with increasing mean BMD and *T*-score values. Linear regressions confirmed a significant proportional bias (*β* = 0.82, *p* < 0.001, for both analyses), indicating that REMS tends to overestimate higher lumbar values and/or underestimate lower ones. Partial explanatory power was observed (*R*^2^ 0.21–0.39), underscoring that REMS only partly captures the variance in DXA measurements. Lin’s CCC demonstrated poor continuous agreement for BMD (total hip 0.46, femur neck 0.43, and lumbar 0.30) and *T*-scores (total hip 0.42, femur neck 0.41, and lumbar 0.25).

**Table 4:** Linear regression and Bland–Altman analyses comparing REMS and DXA measurements at the lumbar spine, total hip, and femoral neck

	**BMD total hip**	**BMD femoral neck**	**BMD total lumbar**
Retained cases (*n*)	147	147	141
Sensitivity			
Specificity			
Cohen *K* (95% CI)			
*R*	0.40	0.29	0.28
*R*^2^	0.39	0.28	0.27
Regression slope	0.93	0.69	1.04
Regression intercept	0.15	0.26	0.11
Mean difference ± DS	0.10 ± 0.13	0.07 ± 0.11	0.15 ± 0.17
	***T*-score total hip**	***T*-score femoral neck**	***T*-score total lumbar**
Retained cases (*n*)	147	147	141
Sensitivity	0.53	0.46	0.62
Specificity	0.76	0.79	0.72
Cohen *K* (95% CI)	0.24 (0.13, 0.34)	0.27 (0.14, 0.39)	0.15 (0.06, 0.24)
*R*	0.32	0.25	0.22
*R*^2^	0.32	0.24	0.21
Regression slope	0.76	0.60	0.88
Regression intercept	0.28	−0.33	1.18
Mean difference ± DS	0.73 ± 0.90	0.56 ± 0.71	0.15 ± 0.17

*CI* confidence interval, *DS* deviazione standard

## Discussion

Our analyses suggest that REMS provides a noninvasive, radiation-free alternative capable of broadly classifying bone status but with moderate sensitivity and limited precision relative to DXA. REMS findings appear most reliable for excluding osteoporosis rather than definitive diagnosis, particularly at the femoral neck and lumbar spine. The modest correlations and agreement metrics reinforce that REMS should currently be considered complementary, with further refinement and validation required for REMS before possible clinical substitution for DXA.

Our findings fit within a heterogeneous body of evidence on DXA–REMS concordance. Notably, the multicenter validation studies underpinning claims of equivalence between REMS and DXA have largely enrolled postmenopausal women or adults without major skeletal comorbidities, consistently reporting very high correlations (Pearson *r* > 0.90) and substantial diagnostic agreement (*κ* > 0.70) [13, 15]. However, when tested in more clinically complex populations, results have been less homogeneous. In cohorts with vertebral osteoarthritis, for example, REMS classified a substantially greater proportion of individuals as osteoporotic compared with DXA (35.1% vs. 9.3%) and identified a higher number of patients with fragility fractures [22, 23]. These findings highlight how degenerative changes and artifacts that inflate DXA readings may mask clinically relevant bone loss, whereas REMS appears less affected by such distortions. In kidney-transplant recipients, reported correlations were *r* = 0.4 at the lumbar spine and *r* = 0.7 at the femoral neck [24]. In our cohort, we likewise observed only moderate DXA–REMS correlations overall and limited categorical agreement (Cohen’s kappa 0.15–0.27), coupled with modest sensitivity (46–62%) but higher specificity (72–79%) and high negative predictive values (0.82–0.93). These results support the view that the DXA–REMS relationship is not uniformly strong across settings, reinforcing the role of REMS as complementary rather than a direct substitute for DXA in current practice.

The findings of this study underscore several clinically relevant considerations for the assessment of bone health in older adults. First, the consistent association between low BMI—likely reflecting intertwined mechanical loading, nutritional, and endocrine pathways—and reduced BMD across skeletal sites highlights the importance of nutritional status and body mass in mitigating skeletal fragility, particularly in underweight older patients who warrant closer surveillance. Large-scale cohort and meta-analytic evidence shows that lower BMI is associated with lower BMD and higher incident fracture risk in older adults [25, 26]. In geriatric populations, undernutrition and sarcopenia frequently co-occur with low BMD and further amplify fracture susceptibility, underscoring the central role of nutritional assessment in bone health [27, 28]. These observations, together with the cross-sectional design, emphasize the need for longitudinal studies to validate REMS-derived parameters and cut-offs and to establish their prognostic value for fracture outcomes.

Second, the greater ability of REMS to capture age-associated skeletal differences deserves careful consideration. Among participants aged ≥ 75 years, REMS consistently showed lower BMD and more negative *T*-scores across all skeletal sites, whereas DXA measurements did not significantly differ between the < 75 and ≥ 75 year groups. This pattern may reflect the well-documented vulnerability of DXA to age-related degenerative artifacts, including osteoarthritis, vertebral deformities, and extraskeletal calcifications, which can artificially inflate BMD values—particularly at the lumbar spine [4, 29, 30]. Importantly, part of the apparent discordance between REMS and DXA, especially in the oldest subgroup, may therefore not solely represent REMS underperformance. Instead, some cases classified as nonosteoporotic by DXA may reflect overestimated bone density due to degenerative changes rather than true preservation of skeletal integrity. In this context, the lower BMD values detected by REMS in adults aged ≥ 75 years may capture age-related bone deterioration that is partially masked by DXA artifacts. By contrast, REMS relies on spectral analysis of raw backscattered ultrasound signals, with algorithms designed to emphasize trabecular bone components and reduce the influence of artefactual spectral patterns [31]. It should be acknowledged that REMS-derived BMD values are not obtained through direct X-ray attenuation, as in DXA, but rather through spectral analysis of raw backscattered ultrasound signals combined with proprietary modeling algorithms. While the primary input derives from the ultrasound signal, demographic and anthropometric parameters may contribute to signal standardization and reference matching. Therefore, REMS outputs should be interpreted as model-assisted estimates of bone status rather than purely physical measurements. In this context, recent methodological discussions have raised important questions regarding the interpretation of REMS-derived BMD values and the extent to which they may be influenced by demographic variables such as age, sex, and BMI [32, 33]. In particular, Chan et al. [31] suggested that a substantial proportion of REMS-derived BMD variability may be explained by demographic and anthropometric parameters integrated within the proprietary algorithmic processing. However, these findings should be interpreted cautiously, as they were derived from exploratory analyses performed in relatively small and selected populations and partly based on simulated modifications of input parameters, which may not fully reflect real-world clinical conditions. Moreover, the association between BMD and demographic or anthropometric characteristics is biologically expected and well established across osteoporosis research [25, 26]. Importantly, our findings suggest that REMS-derived estimates cannot be fully explained by demographic inputs alone. In our cohort, REMS identified age-related skeletal differences that were not detected by DXA, particularly in adults aged ≥ 75 years, a population in whom degenerative artifacts may substantially affect DXA-derived measurements. Furthermore, stratified analyses by age and BMI showed relatively stable REMS–DXA relationships across subgroups, supporting the interpretation that REMS outputs likely reflect a combination of signal-derived skeletal information and algorithmic processing rather than demographic parameters alone.

Nonetheless, our Bland–Altman analyses demonstrated wide limits of agreement, confirming that the two techniques should not be considered interchangeable at the individual level [13, 16]. Even in the presence of a modest mean bias, discrepancies may cross diagnostic thresholds (e.g., from − 2.3 to − 2.7), with direct clinical implications for treatment decisions and follow-up. Therefore, while REMS may provide additional insight into bone status in advanced age, particularly where degenerative changes are prevalent, current evidence supports its role as complementary rather than substitutive in relation to DXA. Taken together with its high specificity and negative predictive value observed in our cohort, these findings suggest that REMS may serve as a pragmatic first-line screening or triage tool in geriatric care, particularly in settings where DXA accessibility is limited or technically challenging (e.g., severe deformities, metallic implants), while recognizing the need for confirmatory DXA assessment when diagnostic uncertainty persists. Several practical aspects support this potential role. REMS is radiation-free, portable, and feasible at the bedside [14, 15], making it suitable for frail older adults or for patients with metallic implants that may interfere with X-ray-based imaging. Its flexible deployment could help streamline pathways and reduce unnecessary DXA referrals by triaging those unlikely to require further testing. At the same time, REMS requires specific operator training and shows greater intra- and interoperator variability than DXA, underscoring the need for standardization and quality control before wider adoption [34]. Overall, these features position REMS not as a replacement for DXA but as a complementary approach within the clinical pathway of bone health assessment in older adults. In addition, recent methodological discussions in the literature have emphasized the importance of clearly defining the conceptual framework of REMS-derived BMD, ensuring transparency in algorithmic processing, and clarifying the extent of its comparability with DXA across diverse clinical settings [35, 36]. These considerations further support a cautious interpretation of REMS outputs and highlight the need for continued external validation and standardization before broader clinical implementation.

### Strengths and limitations

This study presents several strengths. First, the population was comprehensively characterized with detailed demographic, clinical, functional, cognitive, nutritional, and body composition assessments, allowing a multidimensional evaluation of bone health in older adults. Second, the simultaneous use of both DXA, the current gold standard, and REMS enabled a direct and systematic comparison of the two methods, including correlations, regression models, and Bland–Altman analyses. Third, the stratification of results by BMI and age provided novel insights into how these factors modulate the relationship between DXA and REMS, highlighting subgroups in which REMS may perform better. Finally, the study design was embedded within a large national research framework, ensuring standardized procedures and quality control.

Some limitations, however, must be acknowledged. The sample size, although adequate for detecting the observed correlations according to post hoc power analysis, was relatively modest and derived from a single center, which may limit generalizability. The cross-sectional design prevents inference on fracture risk prediction or longitudinal changes in bone status, which are essential for clinical translation. Moreover, potential confounders such as vitamin D status, pharmacological treatments affecting bone metabolism, and inflammatory markers were not assessed and may have influenced BMD results. In addition, the proportion of patients classified as osteoporotic by DXA was relatively small, which may have reduced the statistical power of subgroup analyses and limited the ability to detect potential discrepancies between techniques in this clinically relevant group. REMS is also known to be more operator-dependent than DXA, and although all measurements were performed by trained personnel, variability in technique cannot be fully excluded. Finally, our study did not include older or recently fractured patients, who represent a clinically fragile population with potentially greater measurement variability. In such individuals, discrepancies between REMS and DXA may be even more pronounced and clinically informative, warranting targeted investigations to determine whether REMS maintains comparable performance under these more challenging conditions.

## Conclusions

In summary, this study underscores the ability of REMS to reliably rule out osteoporosis. Its feasibility at the bedside—and in patients with mobility limitations or metallic implants—further highlights its added value in geriatric practice. However, the limited sensitivity, modest positive predictive value, and only fair agreement with DXA indicate that REMS cannot yet replace DXA for diagnostic purposes. Instead, REMS should be considered a first-line screening approach, helping to triage patients who truly require DXA, thereby optimizing healthcare resources and reducing diagnostic delays.

Future work should prioritize longitudinal validation to determine whether REMS measurements predict fracture risk and clinical outcomes. Large multicenter studies are needed to assess reproducibility, quantify operator dependence, and establish standardized cut-offs. Finally, the integration of REMS into screening algorithms in primary care, nursing homes, and community settings deserves exploration, where its portability and safety could have the greatest impact.

## Supplementary Information

Below is the link to the electronic supplementary material.

ESM 1 (PDF 764 KB)

## Data Availability

The data that support the findings of this study are available from the corresponding author upon reasonable request. The data are not publicly available due to privacy and ethical restrictions related to participant confidentiality.
