# Supplementary material for: REMS versus DXA in older adults: diagnostic concordance for osteoporosis in a geriatric outpatient population
Source: Arch Osteoporos. 2026 Jun 11;21(1):88. doi: 10.1007/s11657-026-01723-8 (PMC13260182; doi:10.1007/s11657-026-01723-8)
Supplement: Supplementary file 1 — (PDF 764 KB) [file 11657_2026_1723_MOESM1_ESM.pdf]

Supplementary Table 1. Baseline characteristics of the study population stratified by age groups (<75 vs ≥75 years).

Variable	Age<75 years (n=73)	Age≥75 years (n=75)	p-value
**Age (years)**	70 (67.5;72)	79 (77;83)	<0.001
**Sex, females**	37 (50.7%)	45 (60%)	0.32
**ADL**	6 (5;6)	5 (4;6)	0.003
**CIRS-CI**	1 (1;2)	2 (1;3)	0.002
**MNA**	26.00 (24.50;28.00)	25.00 (23.50;26.50)	0.003
**Total n. drugs**	2 (1;4)	4 (2;6)	0.003
Bone parameters (DXA)			
**BMD lumbar**	1.00 (0.84;1.13)	0.98 (0.83;1.11)	0.79
**BMD total hip**	0.85 (0.72;0.96)	0.80 (0.70;0.91)	0.25
**BMD femur neck**	0.69 (0.59;0.82)	0.67 (0.57;0.75)	0.30
**T-score lumbar**	-0.70 (-1.90;0.35)	-0.60 (-2.00;0.50)	0.78
**T-score total hip**	-1.10 (-1.90;-0.35)	-1.30 (-2.00;-0.60)	0.32
**T-score femur neck**	-1.70 (-2.35;-0.85)	-1.70 (-2.60;-1.10)	0.31
Bone parameters (REMS)			
**BMD lumbar**	0.87 (0.81;0.94)	0.82 (0.75;0.90)	0.002
**BMD total hip**	0.75 (0.68;0.83)	0.68 (0.62;0.78)	0.003
**BMD femur neck**	0.63 (0.56;0.70)	0.58 (0.52;0.67)	0.003
**T-score lumbar**	-1.90 (-2.40;-1.40)	-2.20 (-2.90;-1.60)	0.010
**T-score total hip**	-1.80 (-2.30;-1.30)	-2.20 (-2.70;-1.60)	0.005
**T-score femur neck**	-2.10 (-2.60;-1.55)	-2.40 (-3.00;-1.90)	0.003

Notes: numbers are presented as median (IQR), or count (percentage), as appropriate. *Abbreviations: BMI: Body Mass Index; ADL: Activities of Daily Living; CIRS-CI: Cumulative Illness Rating Scale – Comorbidity Index; MNA: Mini Nutritional Assessment; BMD: Bone Mineral Density.*

Supplementary Fig 1. Correlation between DXA and REMS parameters according to age groups.

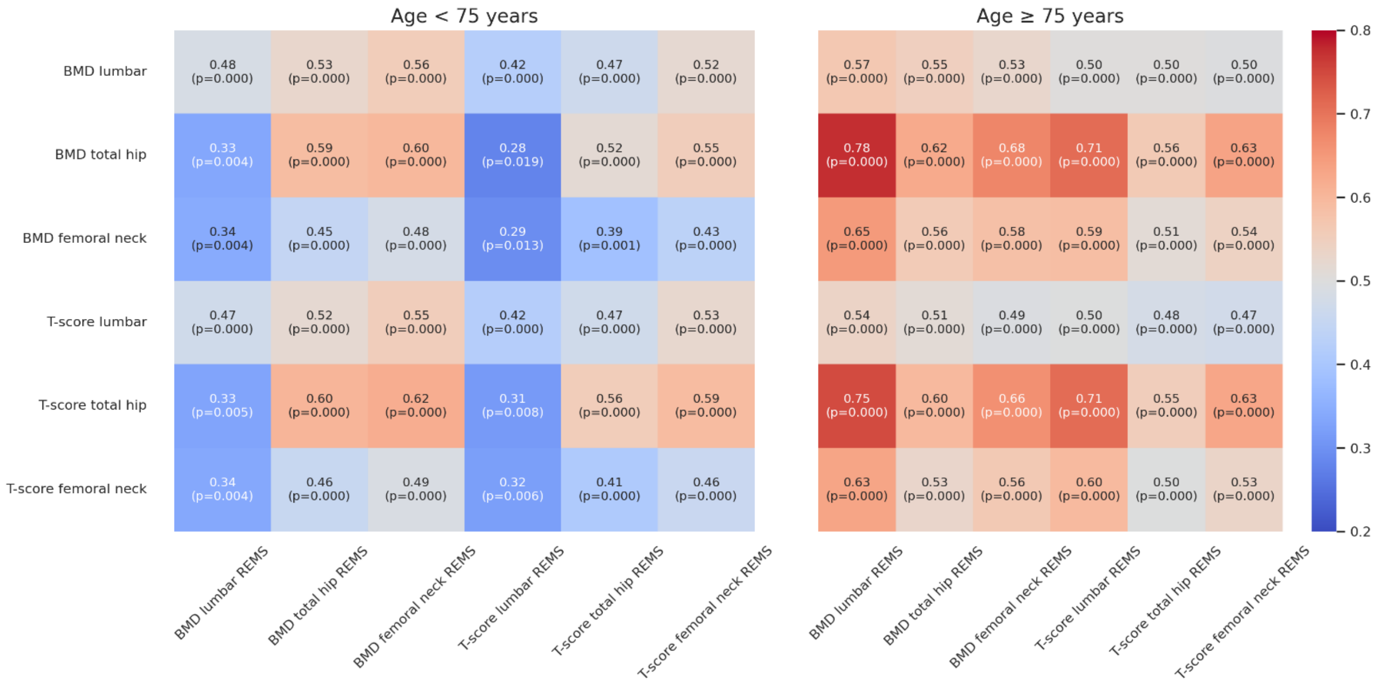

Abbreviations: BMD: Bone Mineral Density.

Supplementary Fig 2. Correlation between DXA and REMS parameters according to sex

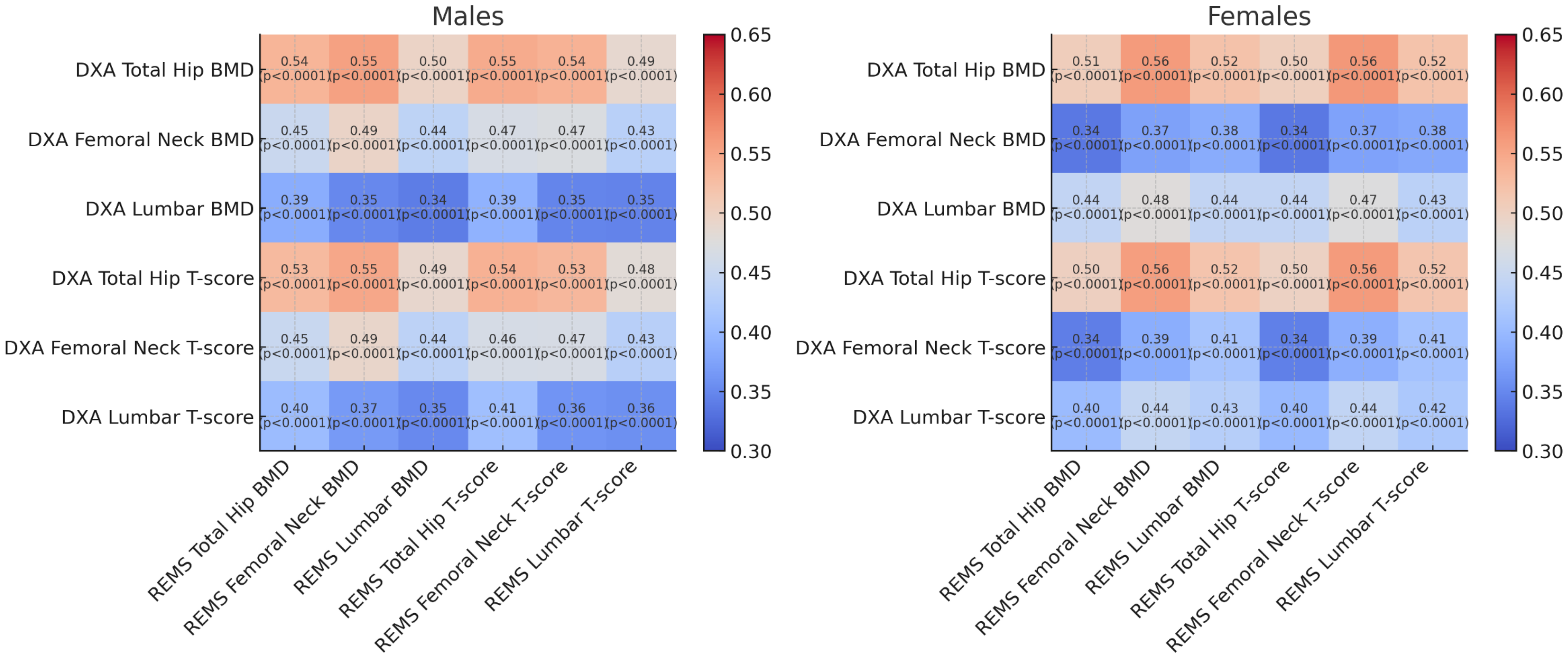

Abbreviations: BMD: Bone Mineral Density.
